# An investigation of oxidant/antioxidant balance in patients with migraine: a case-control study

**DOI:** 10.1186/s12883-019-1555-4

**Published:** 2019-12-14

**Authors:** Mansoureh Togha, Soodeh Razeghi Jahromi, Zeinab Ghorbani, Amir Ghaemi, Pegah Rafiee

**Affiliations:** 1 Headache Department, Iranian Center of Neurological Research, Neuroscience Institute, Tehran University of Medical Sciences, Tehran, Iran; 2 Headache Department, Neurology Ward, Sina University Hospital, School of Medicine, Tehran University of Medical Sciences, Tehran, Iran; 3 Department of Clinical Nutrition and Dietetics, Faculty of Nutrition and Food Technology, Shahid Beheshti University of Medical Sciences, Tehran, Iran; 4 Cardiovascular Diseases Research Center, Department of Cardiology, Heshmat Hospital, School of Medicine, Guilan University of Medical Sciences, Rasht, Iran; 5 Department of Cellular and Molecular Nutrition, School of Nutritional Sciences and Dietetics, Tehran University of Medical Sciences, Tehran, Iran; 6 Department of Virology, Pasteur Institute of Iran, Tehran, Iran; 7 Student Research Committee, (Department and Faculty of Nutrition Sciences and Food Technology), Shahid Beheshti University of Medical Sciences, Tehran, Iran

**Keywords:** Antioxidant defense, Headache, Migraine, Nitrosative stress, Oxidative stress

## Abstract

**Background:**

In recent years, the role of neuroinflammation and oxidative stress in migraine pathogenesis has achieved considerable interest; however, to date findings are equivocal. Thus, the objective of this study was to investigate biomarkers of oxidative stress in episodic and chronic migraineurs (EM and CM patients) and controls.

**Methods:**

Forty-four patients with EM, 27 individuals with CM and 19 age-sex-matched controls were enrolled. After collecting data on demographic and headache characteristics, blood samples were collected and analyzed to detect serum levels of oxidative stress biomarkers (malondialdehyde (MDA) and nitric oxide (NO)); total antioxidant capacity using Trolox equivalent antioxidant capacity (TEAC) assay; and antioxidant enzymes (catalase (CAT), superoxide dismutase (SOD), and glutathione peroxidase-1 (GPx-1)).

**Results:**

Serum levels of CAT and SOD were significantly lower in the CM group than the EM group and controls. However, serum GPx-1 levels of the CM patients were slightly higher than the EM patients and controls (*P*-value≤0.001). CM patients had lower mean TEAC values than EM patients and controls. In addition, serum levels of NO and MDA were significantly elevated among subjects with CM compared to EM and control individuals (*P*-value≤0.001). Pearson correlation analysis revealed negative correlations between the number of days of having headaches per month and serum concentrations of the two antioxidant enzymes CAT (r = − 0.60, *P*-value< 0.001) and SOD (r = − 0.50, P-value< 0.001) as well as TEAC values (r = − 0.61, *P*-value< 0.001); however, there were positive correlations between headache days and serum GPx-1 levels (r = 0.46, *P*-value< 0.001), NO (r = 0.62, P-value< 0.001), and MDA (r = 0.64, P-value< 0.001).

**Conclusion:**

Present findings highlighted that chronic migraineurs had lower total non-enzymatic antioxidant capacity and higher oxidative stress than episodic migraineurs and control individuals. Although more studies are needed to confirm these data, applying novel prophylactic medications or dietary supplements with antioxidant properties could be promising in migraine therapy.

## Background

Migraine has been identified as the leading cause of disability in people under 50 years of age and affects approximately 11% of the adult population of the world [1, 2]. Migraine is categorized into chronic migraine ((CM) defined as experiencing more than 15 days of headache per month with at least 8 days with migraine characteristics or response to Triptans, for at least 3 months) and episodic migraine ((EM) defined as having less than 15 days of migraine headache per month) [3, 4]. If EM patients do not receive proper treatment, their headaches can progress to CM over time [5]. In comparison with EM sufferers, subjects with CM experience greater headache-related disability [6].

Although much effort has been made to elucidate migraine headache pathogenesis, its exact underlying mechanism remains to be understood. To date, different mechanisms have been proposed, including vascular dysfunction, neurogenic inflammation, and activation of the trigeminovascular pathway [7].

Nociceptive information is conducted from the meninges to the brain’s central region and the cortex through the trigeminovascular pathway. In the other direction, trigeminal ganglion cells are responsible for pain sensation through nociceptive receptors in meningeal vasculatures [7]. Headache attacks initiate with stimulation of nociceptive neurons. During this process neuropeptides with vasoactive properties (e.g. calcitonin gene-related peptide (CGRP) and pituitary adenylate cyclase-activating peptide (PACAP)) are released and could result in vasodilatation of arteries, degranulation of mast cells and plasma leakage [7].

The disturbed balance between antioxidant and prooxidant indicators is established as oxidative stress. According to the literature, the important effect of oxidative stress in increasing the risk of a variety of chronic disorders such as cancer, diabetes, hepatic disorders, cardiovascular disease (CVD), and neurodegenerative conditions is well-known [8]. This complicated condition may be a consequence of the disturbances in cellular biochemical pathways that result in increased levels of reactive oxygen and nitrogen species (ROS and RNS), greater vulnerability to oxidative compounds in the environment and reduced antioxidative defense [8]. During oxidative stress, several parameters are involved such as disturbed metabolism of cellular energy phosphate, production of pro-inflammatory mediators and nitric oxide (NO) metabolites, and the presence of high levels of polyunsaturated fatty acids in cell membranes that could predispose the cell to a heightened risk of lipid peroxidation [9]. The antioxidative scavenging system, which is involved in protecting against production of oxidants (e.g. ROS and RNS), consists of enzymatic agents (including superoxide dismutase (SOD), catalase (CAT), glutathione peroxidase (GPx)) and non-enzymatic agents (including glutathione and a number of dietary antioxidants such as vitamin A, C and E) [8]. On the other hand, augmented production of ROS and RNS could damage the intracellular molecules [8]. Oxidation of these molecules, including the proteins and DNA, could lead to dysfunction in cellular structures and organelles and subsequent disruption in receptor function and dysregulation in signaling pathways and the transport system. Emerging animal studies point to the contribution of these events in headache genesis [10, 11]. In this context, measuring levels of oxidative stress indicators can be a noteworthy tool for exploring the relation of oxidant/antioxidant status and pathophysiologic mechanisms of disease development and/or progression [8].

In recent years, the role of neuroinflammation and oxidative stress in migraine pathogenesis has received considerable attention; however, to date findings are equivocal [10, 12–19]. It has been reported that compared to healthy subjects, migraineurs may have higher levels of NO metabolites and malondialdehyde (MDA) that may be produced following an increase in levels of ROS [8, 20]. Also, according to the findings of a meta-analysis of 19 studies on the association between oxidative/nitrosative pathways in migraine, there may be higher levels of oxidative stress indicators and conversely, lower activity of the antioxidant enzyme, SOD, in migraine patients compared to controls [21]. Similar findings have been shown in a number of studies [15, 22–28]. However, there are some studies which have failed to find any differences in the MDA, GPx, SOD and CAT levels of migraine suffers compared to control subjects [9, 18, 25]. Hence, there seems to be a highlighted need to design a study in which the levels of these biomarkers are assessed simultaneously.

Thus, it could be hypothesized that oxidative and nitrosative stress—measured both by serum levels of MDA [8], the most studied biomarker of lipid oxidation end product, and NO [8, 29–31], a well-known oxidant/vasodilator with a pivotal role in migraine pathogenesis—may be present to a greater extent in migraine patients compared with the healthy population while, at the same time, lower antioxidant defense assessed by total non-enzymatic antioxidant capacity and the most-known antioxidant enzymes (CAT, SOD, GPx-1) [8] might also be detected in this population. It could also be assumed that along with an increase in the number of headache days per month, levels of oxidative stress biomarkers may increase whereas the total antioxidant capacity and antioxidant enzymes might decrease. Therefore, we aimed to investigate the serum status of MDA and NO, on the one hand, and total antioxidant capacity, CAT, SOD, and GPx-1, on the other hand, in populations of EM and CM patients compared to controls. Moreover, this research aimed to explore the correlation between the mentioned biomarkers and the number of headache days per month.

## Methods

### Study population

The subjects in both groups were selected following an advertisement asking for voluntary participation in a case-control study on the role of oxidative stress in migraine. Recruitment was from April to June 2018. Episodic and chronic migraine patients were enrolled as the case groups and headache-free individuals were enrolled as controls. The case subjects were recruited from the Sina University Hospital Headache Clinic (a tertiary headache clinic) and the controls were selected from healthy age-sex matched headache-free volunteers from the general population.

The diagnosis of migraine was made following an examination by an expert headache specialist-neurologist according to the International Headache Society Criteria (ICHD-III). Patients having episodic or chronic migraine for at least six months preceding study entry were selected. All patients were in the interictal period and were not experiencing a migraine headache at the time of obtaining blood samples. The eligibility criteria for enrollment in the present study were as follows: being in the age range between 18 and 45 years old; having a body mass index (BMI) between 18.5 and 35 kg/m^2^; not suffering from medication overuse headache (MOH); not having a medical history of inflammatory, infectious, allergic or immune disorders; not having a history of cardiovascular or endocrinological diseases, liver or kidney disorders, or other neurological or chronic diseases such as epilepsy, Parkinson’s disease, multiple sclerosis, or Alzheimer’s disease. Subjects were excluded from the study if they had a history of the above-mentioned disorders or were not willing to sign the informed consent form.

### Headache diaries & visual analog scale (VAS)

At the initial visit, the required information regarding demographic and anthropometric data, medical history, and medications consumed was obtained. Afterward, the collection of blood samples from the participants in the control group was performed. After the examination of migraine patients by the headache specialist-neurologist, the diagnosis of the type of migraine was confirmed. The patients were then instructed on how to fill out a headache diary (designed by senior researcher Prof. M.T. [32]) over the following month. All patients were followed up via weekly telephone calls throughout this month. Using these headache diaries, information on headache features such as the number of headache days, mean severity of headaches, and the number of abortive medications taken was collected. Blood samples were taken simultaneously with the collection of headache diaries at the second visit after 1 month. The Visual Analogue Scale (VAS) scoring system was applied in order to estimate head pain intensity with a score ranging from 0 (having almost no pain) to 10 (the worst possible pain).

### Assessment of serum inflammatory and oxidative stress biomarkers

To detect the serum oxidative stress biomarker levels for the studied patients, blood samples were collected and analyzed for human SOD (ab202410), CAT (ab171572), total non-enzymatic antioxidant capacity using the TEAC assay (ab65329), GPx-1 (ab193767), lipid peroxidation, MDA (ab118970), and NO (ab65328) (all from Abcam), using a commercial enzyme-linked immunosorbent assay (ELISA) kit according to the manufacturer’s instruction. All tests were performed in triplicate.

### Sample size and statistical analysis

Because we did not use a priori sample size calculation, the sample size was of convenience. Post-hoc power analysis was performed applying G*Power software (version 3.1.3) and a comparison of the studied groups with the t-test showed approximately a power of 0.4336, alpha = 0.05 and effect size = 0.5. In order to test the normality distribution of data, a Kolmogorov–Smirnov test was applied. A *P value* of < 0.05 was considered the statistical significance level in all performed analyses. The chi-square test was used to compare the distribution of categorical variables between case and control groups and the independent-sample t-test was performed to examine normal distribution of continuous variables. Analysis of variance (ANOVA) and the Bonferroni post-hoc t-test were used to make comparisons between the two groups of migraine patients and controls for mean values of serum antioxidant and oxidative biomarkers. The Pearson correlation test was applied in order to discover the correlations between headache days per month and oxidative status biomarkers in the migraine patient groups and the correlation coefficients are reported. Statistical analyses were conducted using SPSS 21 (IBM Armonk, NW, US) and GraphPad Prism version 6 (GraphPad Software, La Jolla California USA, www.graphpad.com).

## Results

### Baseline characteristics of the studied population

The baseline characteristics of the studied subjects are presented in Table 1. The mean age of participants in the control group (*n* = 19) was 36 years of age and the mean for migraine patients was 38 (*n* = 71, of whom 44 patients had EM and 27 individuals suffered from CM). About 79% of the subjects in the control group and 86% of the migraineurs were women. No significant differences were noted in age, gender or BMI between the studied groups.

**Table 1:** Baseline Characteristics of the Studied Participants

Variable	Control (n = 19)	Migraineurs (n = 71)	P value
Number (%) of Women	15 (78.9%)	61 (85.9%)	0.45
Age (year) mean (SD)	36 (8)	38 (9)	0.31
Body Mass Index (kg/m2) mean (SD)	25.31 (4.32)	26.16 (4.35)	0.45

*SD*. Standard deviation

### Serum concentration of antioxidants and oxidative stress biomarkers in studied population

**Fig. 1:**
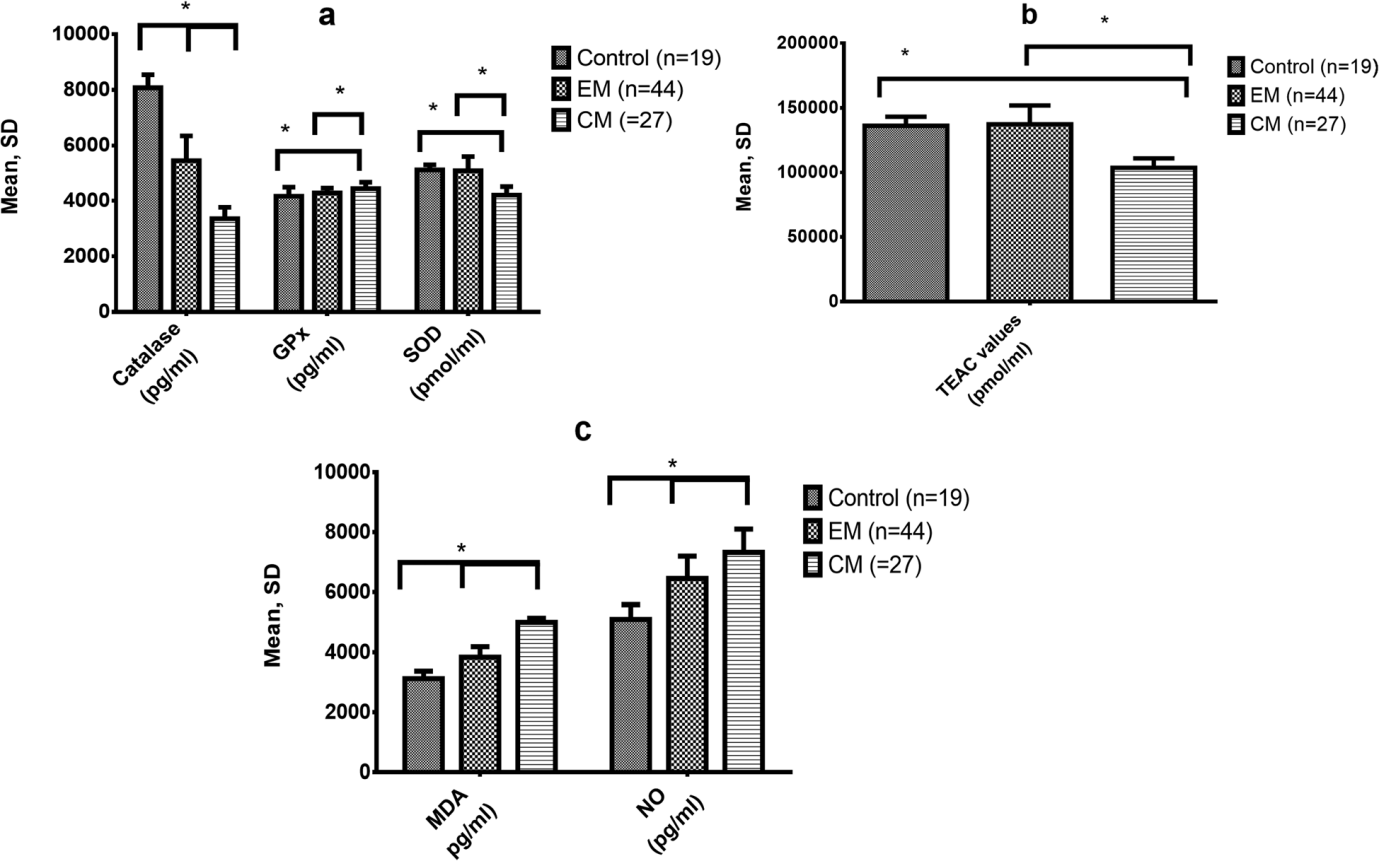
**a** to **c**. Comparison of the mean and standard deviation (SD) of serum oxidative and antioxidant biomarkers in a group of chronic migraineurs (n = 27) and episodic migraineurs (n = 44) compared to healthy controls (n = 19) using analysis of variance (ANOVA) and Bonferroni post-hoc t-test. Significant differences among the compared groups are indicated by *, CAT, Catalase. SOD, Superoxide dismutase. GPx-1, Glutathione peroxidase-1. TEAC, Trolox equivalent antioxidant capacity. NO, nitric oxide. MDA, malondialdehyde

Between-group comparisons of antioxidants and oxidative stress biomarkers revealed that the serum levels of the antioxidant enzymes CAT and SOD were significantly lower in the CM group than in the EM and control groups. Serum concentrations of these enzymes in the EM patients were significantly lower than in controls as well. However, the mean serum GPx-1 level of the CM group was slightly higher than that observed in EM and control subjects (*P*-value≤0.001) (Table 1 and Fig. 1a). Moreover, there was a significant difference in mean serum antioxidant capacity measured by the TEAC assay for episodic migraineurs and controls compared to the CM patients. It was observed that chronic migraineurs had lower TEAC values than EM and control individuals (*P*-value≤0.001) (Table 1 and Fig. 1b). Furthermore, serum levels of NO and MDA were higher among subjects with CM than EM and controls (Table 2 and Fig. 1c) (*P*-value≤0.001).

**Table 2:** Serum inflammatory, oxidative and antioxidant biomarkers in migraine patients compared to healthy controls

Variable	Controls (*n* = 19)		Episodic Migraineurs (*n* = 44)		Chronic Migraineurs (*n* = 27)		*P* value
	Mean	SD	Mean	SD	Mean	SD	
CAT (pg/ml)	8075.95^a^	462.79	5452.14 ^a^	891.98	3354.00 ^a^	414.57	0.000
SOD (pmol/ml)	5117.37 ^a^	183.90	5086.23 ^b^	514.48	4210.63 ^a,b^	308.28	0.000
GPx-1 (pg/ml)	4168.58 ^a^	322.74	4284.23 ^b^	178.71	4441.04 ^a,b^	234.15	0.001
TEAC (pmol/ml)	136,203.68 ^a^	7023.84	137,281.59 ^b^	14,685.31	103,637.41 ^a,b^	7197.49	0.000
NO (pg/ml)	5101.68 ^a^	489.09	6461.23 ^a^	744.32	7329.81 ^a^	775.34	0.000
MDA (pg/ml)	3125.53 ^a^	251.73	3844.93 ^a^	349.75	5005.19 ^a^	138.38	0.000

*CAT*, Catalase. *SOD*, Superoxide dismutase. *GPx-1*, Glutathione peroxidase-1. *TEAC*, Trolox equivalent antioxidant capacity. *NO*, nitric oxide. *MDA*, malondialdehyde. *SD*, Standard deviation

### Headache characteristics

Table 3 presents the comparison of headache characteristics of episodic and chronic migraineurs. The number of consumed analgesics in chronic migraineurs (mean headache days = 23.3 days per month) was significantly higher than EM subjects (mean headache days =9.4 days per month) (mean consumption of analgesic medications per month =10.36 by CM patients vs. 4.15 in the EM group; *P*-value = 0.009). According to the VAS scale, the reported severity of headaches was comparable for both the EM and CM groups (Table 3).

**Table 3:** Comparison of headache characteristics between chronic and episodic patients

Variable	Episodic Migraineurs (*n* = 44)		Chronic Migraineurs (*n* = 27)		*P* value
	Mean	SD	Mean	SD	
Number of headache days per month	9.4	6.0	23.3	6.0	0.000
Headache severity (VAS scale)	7.35	1.52	7.70	2.03	0.410
Number of analgesic medications per month	4.15	3.85	10.36	11.22	0.009

VAS, Visual Analog Scale (VAS)

SD, Standard Deviation

**Fig. 2:**
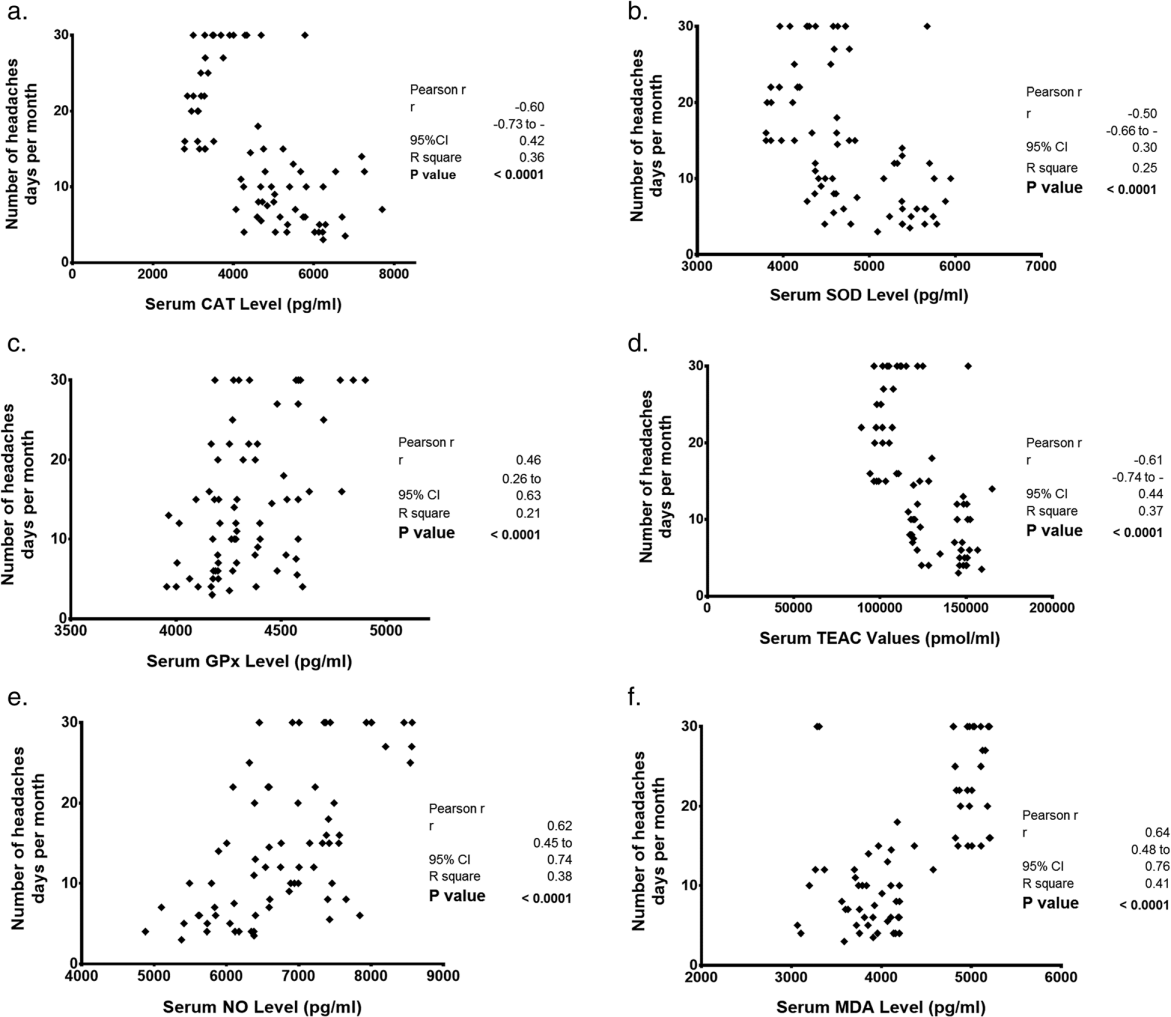
**a** to **f**. The correlation between serum oxidative and antioxidant biomarkers and headache days per month in a group of chronic migraineurs (*n* = 27) and episodic migraineurs (*n* = 44). Pearson correlation test was applied and the correlation coefficients, 95% confidence intervals (CIs) and *P*-values are reported, CAT, Catalase. SOD, Superoxide dismutase. GPx-1, Glutathione peroxidase-1. TEAC, Trolox equivalent antioxidant capacity. NO, nitric oxide. MDA, malondialdehyde

Figure 2 (a-f) depicts the correlation between serum antioxidants and oxidative stress status of migraineurs against their headache days per month. The Pearson correlation analysis revealed significant strong negative correlations between the number of headache days per month and serum concentrations of the two antioxidant enzymes, CAT (r = − 0.60, *P*-value< 0.001) and SOD (r = − 0.50, P-value< 0.001), and the serum antioxidant capacity measured by the TEAC assay (r = − 0.61, P-value< 0.001) among 71 subjects suffering from migraine. There was also a significant medium positive correlation between headache days and serum GPx-1 levels (r = 0.46, P-value< 0.001). Furthermore, significant strong positive correlations were noted for headache days per month and the oxidative stress biomarkers NO (r = 0.62, P-value< 0.001) and MDA (r = 0.64, P-value< 0.001).

## Discussion

In the current study on populations of EM and CM patients and healthy controls, serum levels of MDA (a lipid oxidation end product) and NO (an oxidant vasodilator agent) as biomarkers of oxidative stress were investigated. Further, in order to evaluate antioxidant defense status, three well-known antioxidant enzymes were explored including SOD, catalyzing superoxide (O2−) radical dismutation into O_2_ or H_2_O_2_; CAT, catalyzing H_2_O_2_ conversion to H_2_O and O_2_; and GPx, catalyzing H_2_O_2_ reduction to H_2_O and alcohols via conversion of glutathione oxidation to oxidized glutathione (GSSG) [8]. Total non-enzymatic antioxidant capacity reflecting serum content of non-enzymatic agents with antioxidant features along with dietary antioxidants (such as carotenoids, ascorbic acid, tocopherols, and phenolics agents) [8]. was also determined through TEAC assay. Collectively, the results revealed an imbalance in oxidant/antioxidant status in favor of a higher level of oxidative and nitrosative stress factors in migraine patients than healthy controls. It was shown that, except for GPx-1, serum concentrations of antioxidant enzymes were significantly lower in CM and EM patients than in controls. In addition, it was indicated that chronic migraineurs had lower total non-enzymatic antioxidant capacity than EM and control individuals. While elevated serum levels of NO and MDA were detected among chronic and episodic migraineurs compared to controls.

In line with our results, higher platelet levels of NO metabolites and MDA were detected among migraineurs than in healthy subjects especially during the ictal period in a study by Yilmaz et al. [20]. On the contrary, Bernecker et al. [9] failed to detect significant differences in MDA serum levels for women with migraine compared to controls; it was, however, found that these patients had higher levels than controls of 4-hydroxy-2-nonenal (HNE), another biomarker of lipid peroxidation [9].

MDA is a lipid oxidation end product and could be an acceptable biomarker of oxidative stress and an indicator of increased production of ROS [8]. Based on the relevant literature of animal and human studies, the elevated level of this oxidative mediator has been linked to cortical spreading depression (CSD), one of the main proposed pathological mechanisms in migraine pain initiation particularly in migraine with aura [10, 22, 23, 25, 27, 33–35]. As such, it has been suggested that MDA levels may be attenuated following administration of curcumin, a dietary agent with antioxidant features, in umbilical vein endothelial cells which were considered a model for exploring oxidative stress status in migraine [36].

Additionally, NO is an oxidant vasodilator which could provoke pathways of pain initiation in migraine [8, 30]. As a messenger molecule, NO may be involved in synaptic transmission in neurons and in some of the brain functions including learning, memory, and nociception [29–31]. The present findings on increased levels of NO in both groups of migraineurs (CM and EM) compared to healthy controls is completely in accordance with the current empirical and human evidence that emphasizes the notable role played by NO in migraine pain genesis [29–31]. The stimulating effect of this factor in causing migraine attacks has been studied in depth. This role is highlighted by the elevated production of NO and over-activation of the L-arginine-NO/cyclic GMP signaling pathway in migraine suffers [29–31]. It has been noted that NO could provoke migraine attacks directly by vasodilation through the NO/cyclic GMP signaling pathway in the first phase of attacks and also via increasing CGRP production and activating the nociceptor neurons especially in the trigeminovascular system [29–31]. On the other hand, it has been shown that pharmacological suppression of NO synthesis and/or the signaling pathway could result in decreasing the level of neurogenic vasodilation, neuro-inflammation and treat migraine headaches. NO is believed to be involved in causing neuroinflammation through increasing CGRP and substance P (SP) production in the perivascular nociceptors and stimulation of their activity in trigeminal neurons [29–31].

Additionally, our findings regarding lower serum total non-enzymatic antioxidant capacity, CAT, and SOD levels in migraineurs than controls are partially in accordance with the findings of a meta-analysis on the association between oxidative/nitrosative pathways in migraine. This meta-analysis included 19 studies and reported an increase of about two times in the concentrations of thiobarbituric acid reactive substances (TBARS) in migraineurs compared to controls. While similar to our findings, the activity of the antioxidant enzyme SOD was lower in the studied subjects [21]. Further, in 2014 Aytac et al. conducted an analysis of serum MDA levels and SOD, GPx and CAT activities in 17 controls and 32 migraine suffers (18 patients with deep white matter hyperintensities (WMH)). Similar to the present findings, they showed that the patients had diminished antioxidant response indicated by decreased CAT levels and increased MDA concentrations. They also observed that patients with WMH had lower serum CAT than migraineurs without WMH and healthy subjects [22]. Like the current findings, the higher level of oxidative stress in migraine subjects in comparison with healthy individuals as revealed by augmented values of MDA, NO metabolites, TBARS, lipid peroxidation, total oxidant status (TOS), oxidative stress index (OSI), and plasma lymphocyte DNA damage has also been confirmed by other studies [15, 23–27]. Our results regarding lower TEAC values and two of the antioxidant enzymes (SOD and CAT) are, at least in part, in line with those reported by Bolayir et al. [28], Tripathi et al. [26], and Yigit et al. [27].

However, there are also some researches that have shown conflicting results in this field. For example, in a study by Gupta et al. it was indicated that patients suffering from migraines with aura had higher SOD activity than migraineurs without aura [25]. Furthermore, Tuncel et al. failed to show significant differences in SOD and CAT activities of migraine suffers compared to control subjects [25]. Shukla et al. evaluated the activity of CAT, SOD, and GPx in the blood polymorphonuclear neutrophils of participants but did not observe any significant differences in the activity of these enzymes between migraine cases and control individuals [18].

Surprisingly, present findings on the serum GPx-1 levels revealed that the mean serum GPx-1 levels of the CM group were slightly higher than EM and control subjects. Also, there was a significant medium positive correlation between the number of headache days and serum GPx-1 levels whereas in the research conducted by Bolayir et al. [28] it was demonstrated that migraine patients had lower GPx activities than the patients with tension-type headache and controls. Moreover, Tripathi et al. reported diminished glutathione and glutathione S-transferase (GST) in migraine subjects in comparison with the control group [26]. The possible cause of these results could have been related to the compensatory mechanism in which the levels of GPx-1 may decrease subsequent to the increase of other antioxidant enzymes [37]. Further, it is proposed that as a compensatory process the elevated formation of antioxidant enzymes might occur in response to higher levels of oxidative stress [8]. In this regard, the increased level of GPx in migraineurs observed in the current study may likely be due, at least in part, to reducing SOD and CAT levels in order to preserve balance in the state of oxidative stress [8]. Moreover, it has been pointed out that excess GPx-1 levels, which might be a result of pathological changes in the body or vascular mechanical stress, could lead to adverse effects including the elimination of essential oxidant agents that may cause impaired physiological responses, mitochondrial dysfunction and decreased apoptosis [37].

Thus, although our findings were consistent with most previous reports, there could be some explanations for controversial results obtained in a number of prior researches that make comparison difficult such as the use of different techniques to assess the oxidative/antioxidant balance (e.g. OSI, TOS, total antioxidant status (TAS), ferric reducing ability of plasma (FRAP), TBARS, activities of antioxidant enzymes in erythrocytes or their concentrations in serum).

The positive correlation between the number of headache days and oxidant levels found by Alp et al. in 2010 [16] was partly consistent with the present results. Likewise, Erol et al. showed lower erythrocyte CAT and GPx activities among migraineurs compared to the control group [19]. Our study also showed that increasing oxidative and nitrosative biomarkers and decreasing levels of antioxidant factors were correlated with more frequent headaches which may suggest an inflammatory mechanism in the pathophysiology of EM progression to CM.

Collectively, although the exact mechanisms by which oxidative stress may be involved in head pain pathophysiology has not been clearly defined, there might be some explanations. It is assumed that increased production of NO metabolites and ROS in addition to pro-inflammatory mediators may lead to peroxidation of cell membrane phospholipids, destruction of intracellular molecules (particularly proteins and DNA) and consequently impairment in the normal function of cells [8, 10, 11]. In this respect, emerging evidence has highlighted the contribution of protein and DNA oxidation and subsequent disruption in receptor function, dysregulation in signaling pathways and the transport system to hyperexcitability of cortical neurons, CSD, neurogenic inflammation, and trigeminovascular pathway activation, that ultimately could lead to headache [10, 11]. In addition, mitochondrial biogenesis of the trigeminal nerve may be disrupted in migraine, which could subsequently result in decreased ATP production and increased ROS synthesis [38]. Additionally, CSD, a well-known mechanism in aura generation in migraine, may contribute to increased production of ROS in different parts of CNS including the cortex, meninges and trigeminal ganglia. ROS might induce nociceptive responses directly or through stimulating the secretion of pain mediators in migraine such as CGRP [34, 39]. CGRP, a vasodilator neuropeptide which is known as the main mediator of migraine pain, may be associated with increased formation of pro-inflammatory cytokines and oxidative factors that all are believed to play a pivotal role in pain sensitization [34, 39]. On the other hand, the antioxidant defense consisting of enzymatic or non-enzymatic factors could eliminate ROS and free radicals, protect against oxidative stress induced in migraine [10] and thus alleviate head pain.

### Study strengths, limitations and recommendation for future studies

The strengths of the present study consist of precise diagnosis by an expert headache specialist-neurologist of the type of migraine for both episodic and chronic migraine patients based on ICHD-III criteria; following up migraineurs for a month to record headache features; not including those with MOH; and recruitment of age-sex matched healthy individuals in the control group. However, there were also a number of limitations. First, the case-control design of the present study could be a source of bias in interpreting the findings because this design cannot confirm whether these findings are a cause or a result of migraine headache. Moreover, despite the existence of a variety of confounding factors that could influence the levels of serum oxidative status biomarkers in individuals, we did not include all of them. In addition, evaluating the levels of these markers in the cerebrospinal fluid (CSF) of migraine suffers and exploring the activity of antioxidant enzymes in red blood cells would be most helpful in determining the mechanisms underlying their role in generating migraine pain. Further, the data on time duration from the previous attack and analgesic consumption by migraine patients were not available in this study. These variables could be considered for future research. Importantly, the prevalence of migraine in females is 2–3 times more than in males. Females seem to experience longer, more severe and more disabling headache attacks with higher recurrence risk [40]. Therefore, comparing the oxidative/antioxidant status between females and males who suffer from migraine might be of value in better understanding the pathogenesis of migraine.

Although it should be noted that the findings showing higher levels of oxidative markers in migraine patients may be a cause as well as a result of the disease, novel treatment approaches can be developed based upon these data. Therefore, medications or dietary supplements containing antioxidative and anti-inflammatory agents that can protect against ROS formation may be promising in migraine prophylaxis [10, 11]. Among various dietary approaches, a diet with restricted carbohydrate content like the ketogenic diet may reduce the severity of migraine headaches via improvement of mitochondrial function in the brain, suppression of neuroinflammation, reduction of intracellular ROS production, and negatively affecting CSD and trigeminal activation [41–47]. To date, dietary supplements including riboflavin, coenzymes Q10, melatonin, magnesium, vitamin D and poly-unsaturated fatty acids have been considered likely effective adjuvant therapy options for migraine [11, 48, 49]. Considering these complementary approaches to migraine, prophylactic treatment could be promising. In addition, according to the current state of knowledge, the recently introduced anti-migraine medications that focus mostly on CGRP and its receptors could be effective in migraine relief [50, 51]. Therefore, it seems exploring the effects of drugs targeting CGRP and its receptors in migraine on inflammatory status and oxidative stress biomarkers could further elucidate migraine pathogenesis and shed light on additional mechanisms of the action of these drugs [50].

## Conclusion

The present research revealed an imbalance in oxidant/antioxidant status in favor of higher levels of oxidative biomarkers in migraine patients than healthy controls. Also, it was highlighted that chronic migraineurs had lower total non-enzymatic antioxidant capacity than episodic migraine and control individuals. It was also demonstrated that as the frequency of headache days increased, the level of NO and MDA as biomarkers of oxidative stress went up and the antioxidant defense (as shown by TEAC values, SOD, CAT serum levels) went down. This changing state might be the basis of the progression of migraine from the episodic to the chronic stage. In sum, the current findings seem to further support the relationship of oxidative stress in migraine pathophysiology. Thus, novel prophylactic medications or dietary supplements with antioxidant properties might be promising in migraine treatment generally and also in the prevention of EM becoming CM. Well-designed randomized controlled trials should be conducted in order to assess the effects of the above-mentioned agents on migraine headaches.

## Data Availability

The datasets of the current study are available from the corresponding author upon reasonable request.

## Abbreviations

ANOVA: Analysis of variance
BMI: Body mass index
CAT: Catalase
CGRP: Calcitonin gene-related peptide
CM: Chronic migraine
CSF: Cerebrospinal fluid
ELISA: Enzyme-linked immunosorbent assay
EM: Episodic migraine
FRAP: The ferric reducing ability of plasma
GPx-1: Glutathione peroxidase-1
GSH: Glutathione
ICHD-III: International Headache Society criteria
MAPK: Mitogen-activated protein kinase
MDA: Malondialdehyde
MOH: Medication overuse headache
NO: Nitric oxide
OSI: Oxidative stress index
PACAP: Pituitary adenylate cyclase-activating peptide
RNS: Reactive nitrogen species
ROS: Reactive oxygen species
SOD: Superoxide dismutase
TAS: Total antioxidant status
TEAC: Trolox equivalent antioxidant capacity
TOS: Total oxidant status
VAS: Visual Analogue scale
WMH: White matter hyperintensities
